# A study on the remote sensing estimation and spatiotemporal distribution patterns of aboveground biomass in savanna grasslands of the Yuanmou dry-hot valley

**DOI:** 10.3389/fpls.2025.1648539

**Published:** 2025-09-12

**Authors:** Caiying Chen, Guangxiong He, Haidong Fang, Liangtao Shi, Yongzai Zhuang, Zitian Ding, Junqi Guo, Xuewen Yue, Kunwu Yang, Wenfei Xi

**Affiliations:** ^1^ Faculty of Geography, Yunnan Normal University, Kunming, Yunnan, China; ^2^ Tropical Eco-Agriculture Research Institute, Yunnan Academy of Agricultural Sciences, Yuanmou, Yunnan, China

**Keywords:** biomass, Yuanmou dry-hot valley, machine learning, feature selection, remote sensing

## Abstract

Savanna grasslands in dry-hot valleys play crucial ecological and productive roles, yet accurate estimation of their aboveground biomass (AGB) remains challenging due to rugged terrain, climatic variability, and intensive human disturbances. To address this, we investigated the Yuanmou dry-hot valley in Yunnan, China, by constructing a multi-source dataset that integrated remote sensing indices, topographic factors, meteorological variables, and biodiversity metrics. Three feature selection techniques were applied to identify key predictors, and the selected variables were used to train ensemble machine learning models. Of all tested model combinations, the Random Forest model with Recursive Feature Elimination achieved the highest predictive accuracy, with a coefficient of determination of 0.6975, a root mean square error of 89.3436 g/m^2^, and a mean relative error of 0.7282. The most influential predictors included temperature, latitude, biodiversity indices, and specific spectral bands and vegetation indices. From 2019 to 2024, AGB in the study area exhibited an overall increasing trend and showed increased spatial homogeneity, although low-altitude areas consistently maintained lower biomass due to stronger grazing and land-use pressures. These findings demonstrate the advantages of integrating multi-source variables with machine learning in ecologically heterogeneous regions. The proposed framework effectively reduced redundancy, enhanced sensitivity to ecological drivers, and showed strong adaptability to complex environments. The observed biomass dynamics further highlight the positive effects of ecological restoration policies, while revealing persistent trade-offs between conservation and land use in lowland zones. Overall, this study provides a practical methodological framework for improving the accuracy and applicability of AGB estimation in savanna ecosystems, offering valuable insights for ecological monitoring, policy implementation, and sustainable grassland management.

## 1 Introduction

Aboveground biomass (AGB) of grasslands refers to the total biomass of all aboveground components of grassland vegetation-such as stems, leaves, flowers, and fruits-within a specific time period, typically a growing season or a full year. It reflects the accumulation of plant matter in grassland ecosystems and serves as a key indicator of their carbon sequestration potential and biomass-carrying capacity (Zhao et al., 2014). Accurate estimation of grassland AGB is essential for evaluating community growth status, monitoring successional dynamics, and predicting long-term ecosystem trajectories (Xue et al., 2017; Pan et al., 2024), making it highly significant for ecological research. On the one hand, grassland biomass plays a critical regulatory role in the global carbon cycle, indicating the impact of climate change on grassland ecosystems and revealing feedback mechanisms between vegetation and atmospheric carbon concentrations (Fang et al., 2007; Piao et al., 2009). On the other hand, the quantity of grassland biomass determines the productivity of the ecosystem and directly influences its structural and functional stability (Ni, 2002). Monitoring and assessing grassland biomass therefore holds substantial practical value for applications such as grassland fire risk management, wildlife habitat evaluation, and biodiversity conservation (Hudak and Brockett, 2004; Mutanga and Skidmore, 2004).

Grassland biomass estimation methods are generally categorized into three types based on data sources: field-based measurements, remote sensing-based estimation, and integrated approaches that combine both. Among these, the harvest-based clipping method-a traditional field measurement technique-was widely employed in early studies. This method involves systematically establishing sampling plots, harvesting all vegetation within each plot, and then oven-drying and weighing the samples to estimate regional grassland biomass (Wang et al., 2025). Although the clipping method provides high accuracy for small-scale and easily accessible areas, it has several notable drawbacks, including long sampling cycles, high labor and financial costs, and destructive impacts on the grassland ecosystem. These limitations make it unsuitable for real-time, accurate, and continuous monitoring across large or topographically complex regions (Liu et al., 2006). Since the late 1970s, satellite remote sensing technology has advanced rapidly. Many researchers, both domestic and international, have employed remotely sensed vegetation parameters as predictive variables in biomass estimation models (Zhang et al., 2022). For example, Prince (1991) used NDVI data derived from NOAA satellites to develop a linear regression model and revealed a strong correlation between grassland primary productivity and NDVI. Similarly, Li et al. (2007) constructed biomass estimation models based on the relationships between NDVI, RVI, and vegetation biomass to assess regional biomass levels. Currently, most remote sensing-based biomass estimation approaches rely on *in situ* measurements to establish statistical or regression relationships between remote sensing indicators and ground-observed biomass data. These relationships are then used to develop mathematical models for estimating the AGB of grasslands (Jargalsaikhan et al., 2024). Owing to their efficiency and scalability, these methods have provided a scientific basis for large-scale assessments of grassland growth and have supported the development of sustainable grassland resource management strategies (Lyu et al., 2022; Wang et al., 2022). Consequently, remote sensing-based approaches have become one of the most widely adopted techniques in contemporary biomass estimation research (Liang et al., 2010).

From a modeling perspective, statistical approaches for estimating grassland AGB can be broadly categorized into parametric regression models and non-parametric models. Parametric models are based on the assumption that the data follow a predefined distribution. These models estimate AGB by formulating the relationship between remote sensing variables and field-measured biomass using mathematical equations or functional expressions (Roy et al., 1991; Purevdorj et al., 1998). Parametric models offer advantages such as computational efficiency, structural transparency, and ease of interpretation. However, their predictive performance often declines when faced with complex nonlinear relationships, and they typically exhibit limited generalization capacity. In contrast, non-parametric models make no assumptions about the underlying data distribution. Instead, they learn directly from the data, constructing data-driven relationships through algorithmic mechanisms. With the rapid advancement of machine learning techniques, non-parametric models such as Random Forest (RF), Support Vector Machine(SVM) and Gradient Boosted Decision Trees (GBDT) have been widely applied to AGB estimation. For instance, Zhao et al. (2022) demonstrated that an RF model based on vegetation indices significantly outperformed traditional parametric regression models. Similarly, Wu et al. (2024) compared multiple approaches and found that machine learning algorithms consistently outperformed statistical models in estimating AGB, with RF showing the highest robustness.

Despite substantial progress in grassland AGB estimation, key challenges remain in feature variable selection, model adaptability, and accuracy enhancement. Current remote sensing-based studies often rely on a relatively narrow range of input variables, with modeling processes primarily dependent on traditional vegetation indices. This limits the ability to comprehensively capture grassland growth dynamics in ecologically complex environments. In addition, the performance of different machine learning models varies significantly across geographic regions, and their generalization capabilities and integration efficiency for multi-source data remain inadequate (Zhang et al., 2020). Therefore, it is imperative to develop robust methodologies that can effectively integrate multi-source remote sensing data, optimize feature selection strategies, and improve both estimation accuracy and regional adaptability.

Among various grassland types, Savanna grasslands in dry-hot valley regions are particularly representative. Dry-hot valleys are unique geographical units in southwestern China, shaped by factors such as enclosed topography, deeply incised river valleys, and the foehn wind effect. These factors contribute to a characteristic hot and dry climate, marked by high temperatures, low humidity, and distinct wet and dry seasons (Yang, 2007; Yang et al., 2016). Within this ecological context, a distinct “valley-type Savanna grassland” has developed, dominated by Poaceae herbs along with sparsely distributed trees and shrubs. The plant community typically exhibits three vertical strata-trees, shrubs, and herbaceous plants-showing clear vertical layering and pronounced seasonal dynamics. Common species include *Heteropogon contortus* and *Bothriochloa ischaemum* (herbs), *Vitex negundo* and *Clerodendrum cyrtophyllum* (shrubs), as well as *Phyllanthus emblica* and *Quercus glauca* (trees) (Jin, 1999; Zhang et al., 2021). These grasslands are widely distributed along slopes, alluvial fans, and piedmont terraces within dry-hot valleys, often forming belt-like or patchy clusters. They represent a typical native drought-tolerant vegetation type in the arid regions of southwestern China (He et al., 2009). The Yuanmou dry-hot valley in Yunnan Province serves as a representative study area. In recent years, this ecosystem has faced increasing pressures from climate change and human activities, resulting in severe environmental challenges such as soil erosion and biodiversity loss, thereby classifying it as a typical ecologically fragile region (Zhang et al., 2020).Therefore, developing a high-precision model for estimating the aboveground biomass of Savanna grasslands holds substantial practical significance for grassland management, growth monitoring, and ecological conservation in dry-hot valley regions.

This study focuses on the Savanna grassland in the Yuanmou dry-hot valley, Yunnan, and develops an AGB estimation method that integrates multi-source feature variables, optimized feature selection, and ensemble modeling approaches. Three feature selection algorithms-Spearman’s correlation, Recursive Feature Elimination (RFE), and Lasso regression-are combined with three machine learning models: RF, XGBoost, and GBDT. A comprehensive remote sensing-based AGB estimation model for the Savanna grassland in the Yuanmou dry-hot valley is constructed, and its spatiotemporal distribution patterns are analyzed. Notably, this study represents the first systematic integration of multi-dimensional ecological variables and comparative modeling analysis in the context of sparse tree grasslands in dry-hot valleys. Unlike previous studies that primarily focus on single approaches, such as NDVI combined with RF or SVM, this research further explores feature combination strategies and model adaptability through systematic comparisons. The results demonstrate that the RFE-RF model exhibits strong robustness and generalizability in addressing ecological heterogeneity and the complex terrain characteristic of dry-hot geomorphology, achieving high estimation accuracy and ecological adaptability. These findings provide a scientific basis and data support for dynamic monitoring, grassland resource management, and ecological restoration of typical ecosystems in dry-hot valleys.

## 2 Materials and methods

### 2.1 Technical workflow

**Figure 1:**
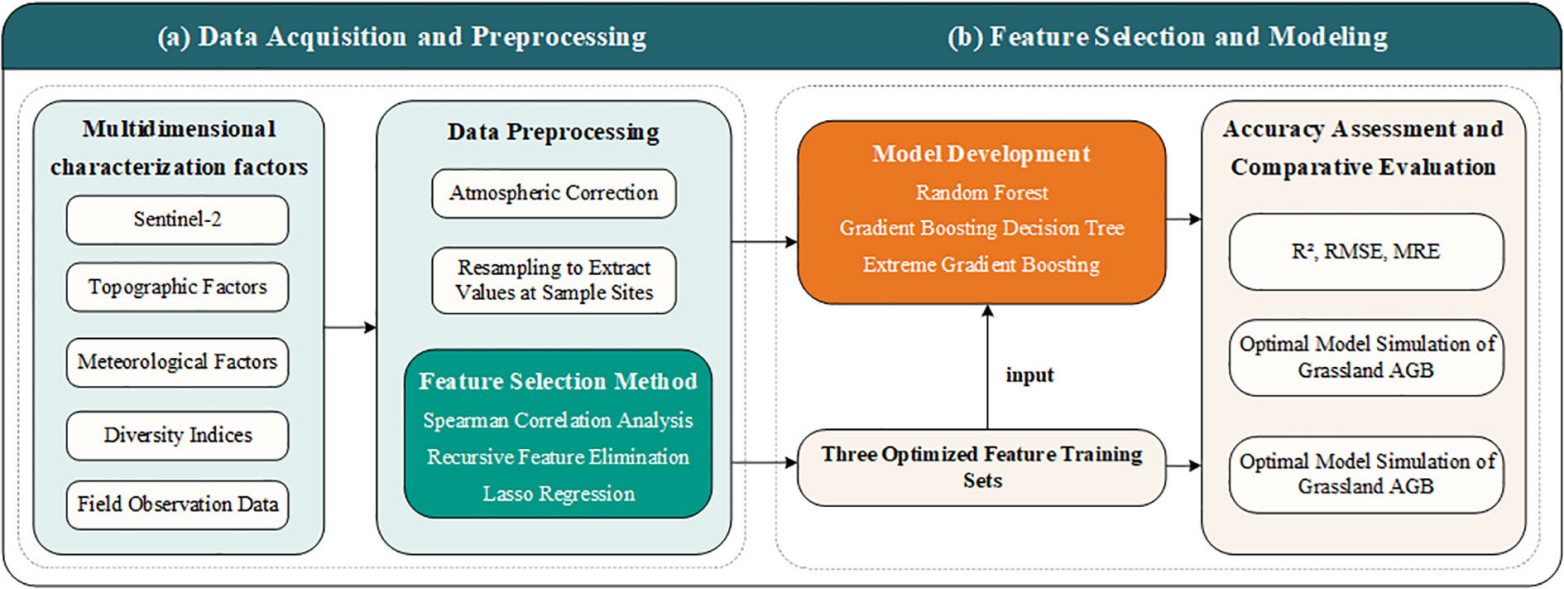
Technical workflow of AGB estimation in savanna grasslands. **(a)** Data Acquisition and Preprocessing, **(b)** Feature Selection and Modeling.

The technical workflow of this study is shown in **Figure 1**. The overall process can be divided into four main steps: (1) Data Acquisition: Integration of vegetation indices, original spectral bands, topographic factors, meteorological variables, diversity indices, and field observation data to construct the initial dataset. (2) Data Preprocessing and Feature Selection: Remote sensing data were subjected to atmospheric correction, resampling, and pixel value extraction. Three feature selection methods were then applied to identify the optimal feature combination. (3) AGB Estimation Modeling: The selected feature sets were input into multiple machine learning algorithms, generating nine AGB estimation models. (4) Model Evaluation and AGB Inversion: Model performance was evaluated using R², RMSE, and MRE, and the best model was chosen for AGB inversion of Savanna grasslands, followed by an analysis of its spatiotemporal distribution patterns.

### 2.2 Study area overview

**Figure 2:**
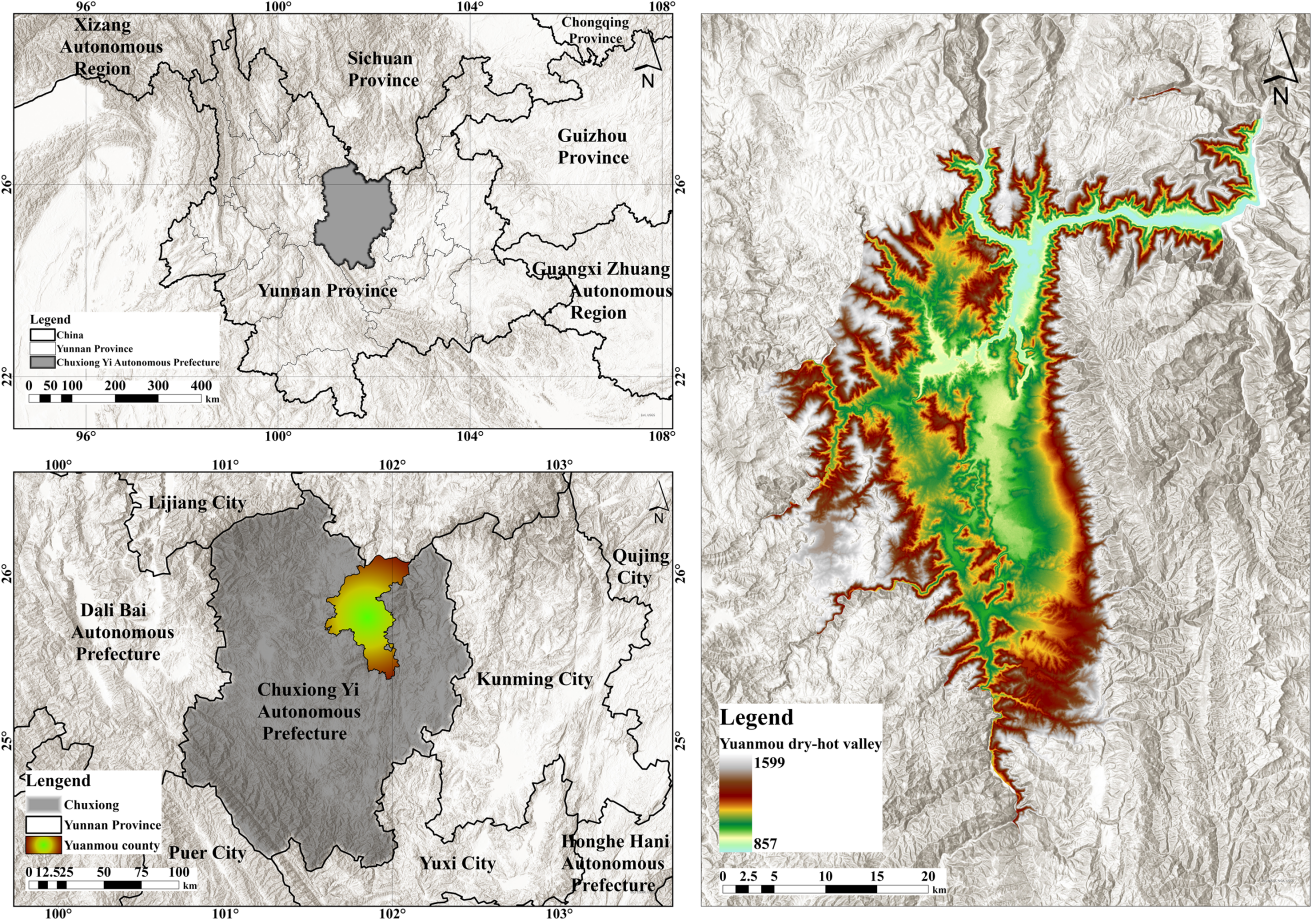
Overview map of the study area.

This study focuses on the grasslands of the Yuanmou dry-hot valley, located in the Chuxiong Yi Autonomous Prefecture of Yunnan Province, China (101°35′–102°06′E, 25°27′–26°04′N). The study area lies in a transitional zone between the Central Yunnan Plateau and the Western Yunnan Plateau (Liu et al., 2024; Zhang et al., 2025), and represents a typical section of the lower reaches of the Jinsha River dry-hot valley (**Figure 2**). It includes all areas within Yuanmou County situated at elevations below 1,600 meters (Zhang et al., 2000; Ou et al., 2015). Geographically, the Yuanmou dry-hot valley exhibits a pronounced north–south orientation, flanked by mountainous terrain on both sides. The valley extends approximately 50 kilometers from north to south and spans about 15 kilometers from east to west, covering a total area of 2,021.47 km² (Zhang et al., 2000). The Yuanmou River, the primary watercourse in the region, flows from west to east through the valley and constitutes a major factor shaping the local geomorphological pattern. Climatically, the region features a typical dry-hot valley climate, characterized by an annual mean temperature of 21.9°C and an average annual precipitation of 615.1 mm. The diurnal temperature variation is significant, whereas annual temperature variation is relatively limited. Summer temperatures often exceed 30°C and can surpass 35°C under extreme conditions, while winter temperatures average around 5°C.

Precipitation in the Yuanmou dry-hot valley exhibits a distinct seasonal pattern. The rainy season, from June to September, accounts for over 90% of the annual total, characterized by intense but short-duration rainfall events. In contrast, the dry season lasts from November to April, featuring minimal precipitation and arid atmospheric conditions, which accentuate the region’s characteristic dry-hot climate. This climatic regime leads to an exceptionally high annual average evapotranspiration of 3,911.2 mm-approximately 6.4 times the annual precipitation-resulting in a pronounced regional moisture deficit (Zhang et al., 2023). Furthermore, both temperature and precipitation demonstrate dramatic variations along elevation gradients, establishing this area as one of the most representative dry-hot valley regions in China. Shaped by the combined influence of environmental and climatic factors, the Yuanmou dry-hot valley has developed an ecosystem dominated by Savanna-type grasslands. These grasslands display a typical vertical stratification of “tree–shrub–grass” and are composed mainly of drought-tolerant, heliophilous herbaceous species. The vegetation is distributed in distinct belts along the valley slopes. In terms of climate, landform, and vegetation composition, the Savanna grasslands in the Yuanmou dry-hot valley exemplify the defining attributes of dry-hot valley ecosystems. They serve as a representative model of grassland-dominated ecological structures in such environments.

### 2.3 Data sources

#### 2.3.1 Field-measured data

##### 2.3.1.1 Ground-based biomass sampling

In accordance with the principles of systematic sampling, comprehensive survey, and targeted verification, AGB data were collected using 1 m × 1 m quadrats within the Savanna vegetation zone below 1600 m elevation in the Yuanmou dry-hot valley during August to October of 2019, 2020, 2021, and 2024. The dominant species in the region include *Bothriochloa pertusa*, *Heteropogon contortus*, and *Phyllanthus emblica*, which exhibit vigorous growth, forming dense grass canopies and substantial litter layers that cover the soil surface. Interspersed among these are various other herbaceous species, contributing to a clearly stratified vertical vegetation structure. Following the sampling methods and technical protocols established by (Fang et al., 2009), multiple 30 m × 30 m randomly located plots were selected across the study area. Within each plot, three independent 1 m × 1 m quadrats were randomly established, with a minimum spacing of 15 meters between them. The selected plots were composed of relatively homogeneous grassland types. All aboveground plant material within each quadrat was clipped at the soil surface. For each quadrat, the sampling date, vegetation height, percent cover, fresh biomass, and species composition (including species names and individual counts) were recorded. The samples were bagged, labeled, and transported to the laboratory, where they were oven-dried at 65°C for 48 hours to determine dry biomass. The mean value of the three quadrats in each plot was used to represent the AGB of that plot. In total, 63, 27, 125, and 114 samples were collected in 2019, 2020, 2021, and 2024, respectively. After data cleaning and quality control, 283 valid samples were retained for use in model development.

Notably, the sample sizes collected across different years showed significant variation, primarily influenced by multiple factors, including the region’s complex topography, harsh climatic conditions, and limited field access-especially during the COVID-19 pandemic. To minimize potential biases caused by these sampling discrepancies, standardized data cleaning procedures and consistent selection criteria were applied during the preprocessing stage. Furthermore, samples from all years were combined into a single dataset, which was then randomly divided into training and testing sets according to a fixed proportion for model validation. This approach has been widely adopted in previous studies and has proven effective in mitigating the effects of sample size imbalance, thereby ensuring the accuracy and generalizability of the model.

##### 2.3.1.2 Diversity indices

Diversity indices are widely used to quantify species richness and the evenness of their distribution, offering essential data support for analyzing the relationship between vegetation structural characteristics and AGB. These indices are extensively applied in ecological research and play a crucial role in AGB estimation (Wang et al., 2024a). In this study, the Simpson index (Simpson, 1949), Inverse Simpson index (Magurran, 2003), Pielou’s evenness index (Pielou, 1966), and Shannon-Wiener index (Shannon and Weaver, 1971) were selected as feature variables and incorporated into the training dataset for the grassland AGB estimation model. All diversity indices were calculated based on field-measured vegetation data.

##### 2.3.1.3 Meteorological data

Meteorological data were obtained from ten monitoring stations established by the Yunnan Meteorological Bureau in Yuanmou County, including temperature and precipitation records from 2019 to 2024, along with the geographic coordinates (latitude and longitude) of each station. Based on these observed data and corresponding elevation information, spatial interpolation was conducted using the ANUSPLIN software. ANUSPLIN is a widely used tool for hydrometeorological interpolation across various spatial and temporal scales because it effectively balances the smoothness and accuracy of the interpolated surfaces. It is particularly well-suited to regions with complex topography (Wang et al., 2024b; Xiao et al., 2023). Therefore, this method was employed in the present study to generate interpolated climate surfaces. The outputs were subsequently processed in ArcGIS for spatial clipping, yielding gridded datasets of annual mean temperature (TEM) and annual total precipitation (PRE) at a spatial resolution of 30 meters for the dry-hot valley region. Finally, climate values corresponding to the year and location of each sampling point were extracted using spatial analysis tools.

#### 2.3.2 Remote sensing data

##### 2.3.2.1 Sentinel-2 data acquisition

The Sentinel-2 satellite is equipped with the MultiSpectral Instrument which captures imagery across 13 spectral bands ranging from the visible to the shortwave infrared region, with a swath width of 290 kilometers. The system operates in a sun-synchronous polar orbit, with a revisit period of 10 days for a single satellite and 5 days when both Sentinel-2A and Sentinel-2B are combined. In this study, Sentinel-2 surface reflectance products with a spatial resolution of 10 meters, provided by the GEE platform, were utilized for data preprocessing. Remote sensing imagery acquired during the grassland growing season (August to October) of 2019, 2020, 2021, and 2024 was selected. Images were filtered based on a cloud cover threshold of less than 10%, yielding a total of 360 valid Sentinel-2 scenes. To capture optimal vegetation conditions, cloud masking was performed using the QA60 band and the cloudMask function available in GEE. Subsequently, the QualityMosaic function was applied to generate composite images that represent the highest-quality pixels across the time series. Two categories of remote sensing features were extracted from the imagery: (1) original spectral bands and (2) vegetation indices. These feature sets were then incorporated into the model development process for estimating grassland aboveground biomass.

##### 2.3.2.2 Original spectral bands

The Sentinel-2 satellite imagery includes 13 multispectral bands, each designed for specific observational purposes. In this study, Bands B1 through B12 were extracted from the Sentinel-2 data based on their respective spectral characteristics and functional relevance for vegetation monitoring and analysis. Detailed information on each band is provided in **Table 1**.

**Table 1:** Sentinel-2 spectral band information.

Band	Wavelength range (µm)	Spatial resolution (m)	Description
B1 (Aerosol)	0.433 - 0.453	60	Used for atmospheric correction, aerosol monitoring, and coastal zone studies
B2 (Blue)	0.459 - 0.525	10	Used for vegetation reflectance monitoring and land cover classification
B3 (Green)	0.543 - 0.578	10	Used for healthy vegetation monitoring and water turbidity analysis
B4 (Red)	0.650 - 0.680	10	Used for vegetation index calculation; sensitive to vegetation absorption and reflectance
B5 (Red Edge 1)	0.698 - 0.713	20	Used for vegetation health monitoring; sensitive to chlorophyll content variation
B6 (Red Edge 2)	0.733 - 0.748	20	Used for monitoring vegetation structural characteristics
B7 (Red Edge 3)	0.773 - 0.793	20	Used for vegetation health assessment
B8 (NIR)	0.785 - 0.899	10	Used for vegetation indices; suitable for vegetation cover and productivity assessment
B8A (Narrow NIR)	0.855 - 0.875	20	Used for more precise vegetation health monitoring
B9 (Water Vapor)	0.935 - 0.955	60	Used for water vapor content monitoring and atmospheric correction
B10 (Cirrus)	1.360 - 1.390	60	Used for cirrus cloud detection and image quality improvement
B11 (SWIR 1)	1.565 - 1.655	20	Used for soil moisture monitoring, crop classification, and atmospheric correction
B12 (SWIR 2)	2.100 - 2.280	20	Used for surface mineral and soil information extraction; suitable for disaster monitoring

##### 2.3.2.3 Vegetation indices

Vegetation indices (VIs) are derived by linearly or non-linearly combining different spectral bands to quantitatively or qualitatively represent vegetation coverage and growth conditions. They play a vital role in evaluating grassland productivity and ecological functions. As a widely applied remote sensing technique, VIs are extensively used in vegetation cover monitoring, growth condition assessment, and tracking ecological restoration or degradation processes. Numerous studies have shown significant correlations between vegetation indices and grassland aboveground biomass (Bao et al., 2008; Jin et al., 2014). In this study, four spectral bands from Sentinel-2 imagery-blue (B2), green (B3), red (B4), and near-infrared (B8)-were selected for vegetation index calculation. Based on the spectral characteristics and suitability of each index, a total of 30 vegetation indices, including NDVI, RVI, and EVI, were computed. These indices were used to evaluate vegetation cover status and monitor ecosystem dynamics.

##### 2.3.2.4 DEM data acquisition

The 30-meter resolution Digital Elevation Model (DEM) and administrative boundary vector data were obtained from the Resource and Environment Science and Data Center of the Chinese Academy of Sciences (https://www.resdc.cn/Default.aspx). The DEM data were preprocessed using ArcGIS 10.2 through mosaicking and spatial clipping to produce the geospatial datasets required for this study. Based on the DEM of the Yuanmou Dry-Hot Valley, a set of terrain-related physical parameters-including latitude, elevation, slope, aspect, and surface roughness-were derived using spatial analysis tools in ArcGIS 10.2.

### 2.4 Variable selection

A total of 53 initial modeling variables were constructed, including 12 original remote sensing spectral bands, 30 vegetation indices, 4 biodiversity indices, 5 topographic factors, and 2 meteorological variables. To effectively eliminate redundancy among variables, mitigate multicollinearity, and improve the predictive performance of the model, three feature selection methods were applied: Spearman correlation analysis (Cohen, 1988), RFE (Adorada et al., 2018), and Lasso regression (Hastie et al., 2001). The specific procedures and selection criteria for each method are outlined as follows:

#### 2.4.1 Spearman correlation analysis

This method was used to evaluate the monotonic relationships between each variable and the ground-observed AGB. Spearman’s rank correlation coefficient (ρ) was calculated for each variable in relation to the measured AGB, and the absolute values of the coefficients were ranked in descending order. Variables with weaker correlations were progressively excluded to form a preliminary feature subset for subsequent modeling. Instead of using a fixed correlation coefficient threshold, a feedback-based approach was adopted, whereby the feature subset was dynamically adjusted according to the variation trend of the root mean square error (RMSE) in machine learning models constructed with different variable combinations. The final optimal feature subset was determined based on the variable combination that yielded the lowest RMSE.

#### 2.4.2 Recursive feature elimination

This method employed a Random Forest regressor as the base estimator and implemented Recursive Feature Elimination with Cross-Validation (RFECV) to perform feature selection. In each iteration, the least important feature-identified based on feature importance scores-was eliminated using a step size of one. Concurrently, 10-fold cross-validation was applied to evaluate model performance using the negative mean squared error (neg-MSE) as the assessment metric. The optimal number of features and the corresponding feature subset were selected based on the configuration that yielded the lowest RMSE. This method does not require the manual setting of an importance threshold, thus offering strong adaptability and robustness in model construction.

#### 2.4.3 Lasso regression

Lasso regression incorporates an L1 regularization term to simultaneously perform variable selection and coefficient shrinkage. In this study, the LassoCV module was utilized to automatically identify the optimal regularization parameter α (alpha) via 10-fold cross-validation, upon which the final Lasso model was constructed. Only features with non-zero regression coefficients were retained, enabling sparse modeling. The optimal α value determined in this study was 281.95. By penalizing the coefficients of less informative features, Lasso regression drives them toward zero, thereby achieving dimensionality reduction while preserving the most predictive variables.

The three feature selection methods were applied independently, with the selected variable subsets used to construct modeling schemes for different input combinations. By comparing the estimation accuracy and stability of models across these feature configurations, we evaluated the applicability and effectiveness of each method in feature extraction.

The modeling variables used in this study encompass data from multiple dimensions, each complementing the others in terms of ecological indicators. Remote sensing raw bands and vegetation indices directly reflect the spectral characteristics and growth status of vegetation. Numerous studies have confirmed their strong correlation with AGB, particularly in capturing spatial variations in grassland biomass. Terrain factors, such as slope, aspect, and surface roughness, influence plant growth by modulating solar radiation, water distribution, and runoff processes, thus shaping the microclimatic conditions that support vegetation development. Meteorological factors, including annual mean temperature and precipitation, serve as dominant environmental drivers at the macro scale, directly impacting net primary productivity and vegetation carbon accumulation rates. Additionally, diversity indices reflect the complexity of community structure, including species richness, evenness, and ecological stability. These are critical indicators for assessing the resilience and recovery capacity of grassland ecosystems, particularly in ecologically sensitive areas such as dry-hot valley regions. In the Yuanmou dry-hot valley, characterized by an arid climate, fragmented terrain, and significant ecological transitions, relying on a single data source or variable type often fails to fully reveal the mechanisms behind AGB formation. Therefore, the integration of multi-source variables not only enhances the model’s ability to explain spatiotemporal biomass variations but also strengthens its adaptability and responsiveness in the context of ecological heterogeneity.

### 2.5 Machine learning model construction

This study employed machine learning algorithms as the primary predictive approach, utilizing three ensemble models: RF, XGBoost, and GBDT. By combining these three machine learning methods with three feature selection algorithms, a total of nine remote sensing models for above-ground biomass estimation were developed. For each model combination, a grid search was performed to optimize key hyperparameters, with the goal of achieving optimal predictive performance.

#### 2.5.1 RF model construction

The RF algorithm, developed by Leo Breiman and Adele Cutler, is an ensemble learning method that constructs multiple decision trees and obtains the final prediction by averaging the outputs of all trees (Reinermann et al., 2020). In this study, grid search was employed to optimize the model parameters on training datasets constructed using three different feature selection algorithms. Among the key parameters, n_estimators refers to the number of decision trees used in the ensemble, where increasing the number generally improves model accuracy but excessively large values may reduce computational efficiency. max_depth represents the maximum depth of each decision tree, i.e., the maximum number of layers from the root node to the leaf node; this parameter controls the complexity of the trees, with deeper trees potentially causing overfitting and shallower trees possibly leading to underfitting. The optimal parameters of the RF model are summarized in **Table 2**.

**Table 2:** Important hyperparameters of the RF Model.

Parameter	Spearman correlation	RFE	Lasso regression
n_estimators	200	150	200
max_depth	None	14	None

In Python 3.8, the Random Forest Regressor from the scikit-learn (sklearn) library was employed to construct remote sensing estimation models for grassland aboveground biomass using three optimized training datasets derived from different feature selection algorithms. The independent variables consisted of features selected by these algorithms, while the measured biomass data served as the dependent variable. The model randomly partitioned 90% of the input data for training and reserved the remaining 10% as a test set to evaluate model accuracy.

#### 2.5.2 GBDT model construction

GBDT, proposed by Friedman in 2001, is designed to enhance predictive performance by integrating multiple weak learners. This approach iteratively optimizes the model via residual fitting, progressively correcting errors to improve its fitting capability (Wang et al., 2023). Grid search was used to tune parameters for the three feature selection algorithms. Among these parameters, the learning rate controls the contribution of each individual tree to the final prediction, while n_estimators specifies the number of decision trees. The optimal GBDT model parameters are summarized in **Table 3**.

**Table 3:** Important hyperparameters of the GBDT model.

Parameter	Spearman correlation	RFE	Lasso regression
learning_rate	0.1	0.3	0.1
n_estimators	50	50	200

The GBDT model was implemented using the Python GBDT library. A remote sensing model for grassland above-ground biomass estimation was developed based on three optimized feature-selected training datasets and measured biomass data. These three optimized feature sets were used as independent variables, while the measured biomass values served as the dependent variable. The model randomly selected 90% of the input data to form the training set, with the remaining 10% used as the test set to assess model accuracy.

#### 2.5.3 XGBoost model construction

XGBoost, developed by Tianqi Chen in 2014 as an enhancement of the GBDT algorithm, iteratively trains a new decision tree at each iteration by optimizing the gradient of the prediction errors from the previous model, thereby progressively reducing the overall error (Chen and Guestrin, 2016). Grid search was employed to optimize the parameters of XGBoost to improve its predictive performance in AGB estimation. The learning_rate controls the step size of each iteration, while n_estimators denotes the number of weak learners. The optimal parameters of the XGBoost model are summarized in **Table 4**.

**Table 4:** Important hyperparameters of the XGBoost model.

Parameter	Spearman correlation	RFE	Lasso regression
learning_rate	0.1	0.1	0.3
n_estimators	50	50	100

The XGBoost model was implemented using the Python XGBoost library. Remote sensing estimation models for grassland aboveground biomass were developed based on three optimized feature-selected training datasets and measured biomass data. These three optimized feature sets served as independent variables, while the measured biomass values were treated as the dependent variable. The model randomly selected 90% of the input data to constitute the training set, with the remaining 10% used as the test set to evaluate model accuracy.

### 2.6 Model accuracy comparison and evaluation

Model accuracy was evaluated using the coefficient of determination (R² Equation 1), root mean square error (RMSE, Equation 2), and mean relative error (MRE, Equation 3) (Zhou et al., 2023; Li et al., 2024). The corresponding calculation formulas are provided below:


(1) $$R^{2}=1-\frac{\sum_{i=1}^{n}{\left(y_{i}-\hat{y_{i}}\right)}^{2}}{\sum_{i=1}^{n}{\left(y_{i}-\bar{y_{i}}\right)}^{2}}$$

(2) $$RMSE=\sqrt{\frac{\sum_{i=1}^{n}{\left(y_{i}-\hat{y_{i}}\right)}^{2}}{n}}$$

(3) $$MRE=\frac{1}{n}\sum_{i=1}^{n}|\frac{y_{i}-{\hat{y}}_{i}}{y_{i}}|\times 100\%$$

In the formulas, $y_{i}$ represents the observed value of the i -th sample, $\hat{y_{i}}$ denotes the predicted value of the i -th sample, $\bar{y}$ is the mean of the observed values, and n is the total number of samples. The R² indicates the goodness of fit between the model predictions and the observed values, with values closer to 1 representing better model performance. The RMSE quantifies the square root of the average squared differences between predicted and observed values; lower RMSE values indicate smaller prediction errors and improved model fit. The MRE expresses the relative difference between predicted and observed values.

## 3 Results and analysis

### 3.1 Characteristics of aboveground biomass in the savanna grasslands of Yuanmou dry-hot valley

Statistical analysis was performed on AGB sample data collected within the study area from 2019 to 2024, with the results summarized in **Table 5**. Overall, significant differences were observed in the mean, maximum, and minimum AGB values across different years, reflecting considerable spatiotemporal variability in vegetation growth conditions within the region. Notably, the average AGB in 2020 was 305.24 g/m², markedly higher than in other years, whereas the mean AGB in 2019 was comparatively low at 53.13 g/m².

**Table 5:** Statistical summary of measured AGB values from sample plots in 2019–2021 and 2024.

Statistical indicators	Year 2019	Year 2020	Year 2021	Year 2024
Number of samples	63	27	125	114
Mean (g/m²)	53.13	305.24	172.62	187.82
Maximum (g/m²)	181.49	607.36	578.60	814.43
Minimum (g/m²)	0.4	89.79	22.98	19.89
Standard error	5.024	25.35	9.38	14.94

At the plot scale, the distribution of maximum and minimum AGB values reflects differences in management intensity and ecological conditions among sample sites. In 2024, the maximum AGB reached 814.43 g/m², indicating the presence of plots with high biomass, while the minimum AGB was only 19.89 g/m², revealing low-biomass plots likely influenced by environmental factors, which contributed to substantial variability in AGB across plots. Additionally, the maximum AGB in 2020 was 607.36 g/m², significantly higher than in 2019 and 2021, whereas the minimum was 89.79 g/m², indicating the persistence of locally degraded plots. Spatial heterogeneity is a major factor driving AGB differences both temporally and spatially. The standard error of AGB in 2021 was 9.38 g/m², markedly lower than in other years, suggesting more uniform vegetation growth across plots that year. Conversely, the standard error in 2020 was relatively high at 25.35 g/m², reflecting pronounced variability in growth conditions among the sample plots.

### 3.2 Feature selection results

Important variables were selected using three feature selection methods: Spearman correlation analysis, RFE, and LASSO regression. The feature selection results are summarized in **Table 6**.

**Table 6:** Optimal variable selection results from three feature selection algorithms.

Method	Selected variables
Spearman correlation analysis	RDVI、Temperature、EVI、B12、GNDVI、NDWI、CVI、GRVI、GCI、B8、B7、ARVI、RVI、B8A、MSR、PRI、MNLI、NDVI、EVI2、TSAVI、GSAVI、OSAVI、SAVI、TNDVI、MSAVI、MSAVI2、B6、Simpson、RGBVI、B9、B3、B5、B4、B2、B 1
RFE	N、Simpson、Shannon.Wiener、Pielou、B1、B5、B11、GRVI、Temperature
Lasso regression	elevation、TVI2、B2、B5、B6、B7、B8A、B9、B12、TVI、Precipation

According to the selection results, Spearman correlation, RFE, and LASSO regression identified 35, 9, and 11 optimal feature variables, respectively. The features selected by the RFE method were relatively balanced across different categories, including topographic factors, diversity indices, remote sensing indices, and climatic factors. Among them, latitude (N) and temperature were identified as key topographic and climatic variables influencing the variation in AGB of grasslands in the dry-hot valley region. Although latitude exhibits relatively small variation within the study area, it remains an important indicator of local microclimates and vegetation types, affecting plant growth potential and community distribution patterns. The Yuanmou Dry-Hot Valley is characterized by high temperatures, low precipitation, and large diurnal temperature variations. Temperature directly influences plants’ photosynthetic efficiency, transpiration rate, and water use efficiency, thereby exerting a direct effect on grassland aboveground biomass. The Simpson, Shannon-Wiener, and Pielou indices were used to quantify species richness, evenness, and stability within the vegetation communities, reflecting the complexity of ecosystem structure. In this harsh climatic environment with significant human disturbance, the stability and adaptability of community structure play a crucial role in shaping AGB. Additionally, spectral bands B1, B5, B11, and the Green-Red Vegetation Index (GRVI) were identified as important factors affecting grassland AGB. Spectral information has consistently been a key feature in AGB inversion, showing strong correlations with biomass.

### 3.3 Comparison of model accuracy for grassland aboveground biomass estimation

AGB estimation models were developed by combining the optimal feature sets derived from three feature selection methods-Spearman correlation, RFE, and LASSO regression-with three machine learning algorithms: RF, XGBoost, and GBDT. This resulted in a total of nine model combinations. The performance of each model on the test dataset is presented in **Table 7**.

**Table 7:** Accuracy evaluation of AGB estimation under different algorithm combinations.

Feature selection algorithm	Evaluation metrics	RF	XGBoost	GBDT
Spearman correlation	R^2^	0.6264	0.5773	0.6591
	RMSE	99.2946	105.6116	94.8422
	MRE	1.1172	0.9415	1.1064
RFE	R^2^	0.6975	0.6940	0.6487
	RMSE	89.3436	89.8636	96.2793
	MRE	0.7282	0.5178	0.7166
Lasso regression	R^2^	0.5875	0.5254	0.6953
	RMSE	104.3353	111.9124	88.1884
	MRE	1.3897	1.3793	0.9650

Overall, the RF model exhibited the greatest adaptability to different feature selection methods, achieving consistently high predictive accuracy with both RFE and Spearman’s correlation-based feature sets. In contrast, the SVM model showed higher sensitivity to the choice of feature selection method, with notably poorer performance when using features selected by the LASSO. The XGBoost model performed best when combined with the RFE-selected features. Among the nine model combinations evaluated, the RFE-RF combination achieved the best overall performance, with the highest goodness-of-fit (R² = 0.6975). Therefore, this model was selected to estimate grassland aboveground biomass in the Yuanmou Dry-Hot Valley region.

### 3.4 Spatiotemporal distribution patterns of grassland biomass

#### 3.4.1 Temporal variation

**Figure 3:**
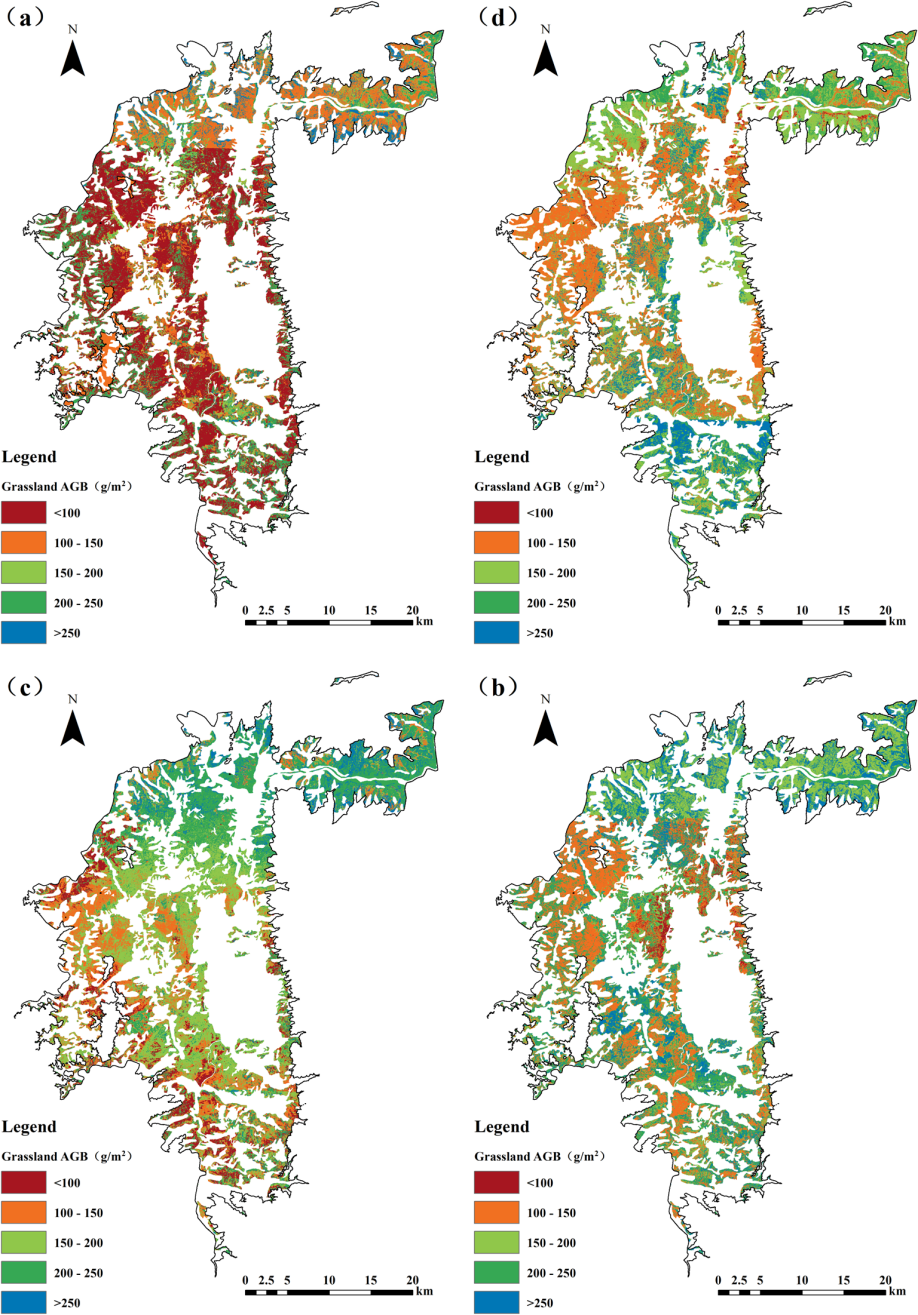
Estimated aboveground biomass of grasslands: **(a)** 2019; **(b)** 2020; **(c)** 2021; **(d)** 2024.

**Figure 3** illustrates the estimated AGB of savanna grasslands in the Yuanmou dry-hot valley, derived using the RFE-RF model. A comparative analysis of the spatial distribution maps from different years (2019–2024) reveals a sustained upward trend in AGB and a progressively more spatially balanced distribution. This trend reflects a clear spatiotemporal response in grassland productivity, indicating an ongoing ecological recovery process driven by the combined influence of anthropogenic activities and natural factors within the dry-hot valley ecosystem. In 2019 (**Figure 3a**), the initial year of the study period, AGB levels were generally low. Red and orange zones were widely distributed across the central, southwestern, and northwestern regions of the study area, indicating that AGB values were predominantly below 150 g/m², with a considerable proportion falling below 100 g/m². These low values signify sparse vegetation cover and limited biomass accumulation, suggesting that ecosystem functioning was substantially constrained. Regions with relatively higher AGB were sparsely distributed, mainly confined to the eastern margin and specific river terrace areas. This spatial pattern is indicative of a typical degradation structure, characterized by “low-value dominance with isolated high-value patches.” In 2020 (**Figure 3b**), a slight overall increase in AGB was observed, along with a noticeable expansion in green and cyan zones. The area corresponding to moderate AGB values (150–250 g/m²) expanded, indicating initial signs of productivity recovery and a shift toward moderate biomass levels. Compared to 2019, regions with low AGB (<100 g/m²) were significantly reduced, suggesting that early-stage ecological restoration interventions-such as grazing exclusion, reduced stocking rates, and improvements in local environmental conditions-had begun to exert a measurable positive effect on grassland conditions. However, patches of low biomass persisted, particularly in areas characterized by complex topography, indicating that the full restoration of ecosystem functioning had not yet been achieved. In 2021 (**Figure 3c**), the spatial distribution of AGB experienced a significant shift, marked by a pronounced enhancement in grassland productivity. Green, cyan, and blue areas expanded rapidly throughout the study area, indicating a substantial increase in regions where AGB exceeded 200 g/m². Notably, areas with exceptionally high biomass values (>250 g/m²) emerged for the first time as contiguous patches, primarily concentrated in the northwestern, eastern, and southern margins. This marked increase reflects a notable improvement in vegetation growth conditions, likely driven by the sustained implementation of ecological restoration measures, and signals a significant strengthening of ecosystem functioning. By 2024 (**Figure 3d**), this recovery trajectory had become further consolidated. Medium- to high-biomass zones (AGB between 200–250 g/m² and above) were widely distributed across the entire region, suggesting the emergence of a more spatially homogeneous and ecologically stable grassland structure. In contrast, low-biomass areas (<100 g/m²) became increasingly sparse, occurring only sporadically in central terrace zones and in regions with steep or rugged topography. Degraded patches were significantly diminished in both size and frequency. Overall, the 2024 AGB distribution exhibited a successional pattern characterized by the “dominance of medium-to-high biomass values with only marginal persistence of low-biomass zones,” indicating that the savanna grassland ecosystem had entered a relatively stable phase of ecological recovery.

Taken together, from 2019 to 2024, the AGB of savanna grasslands in the Yuanmou dry-hot valley displayed a significant upward temporal trend. The spatial configuration evolved from an initial pattern characterized by “low biomass and fragmentation” to a more advanced stage defined by “medium-to-high biomass and spatial continuity.” This transformation was especially evident in 2021 and 2024, when grassland productivity showed remarkable improvement. These findings suggest that, under the constraints of a dry-hot climatic regime, the regional savanna grassland ecosystem exhibits a notable degree of resilience and intrinsic self-restoration capacity. The observed positive trajectory can be attributed to the synergistic influence of improved climatic variability, the phased implementation of grassland management policies, and the long-term enforcement of ecological restoration initiatives-all of which have collectively contributed to the enhancement of ecosystem recovery and stability.

As shown in **Table 8**, the maximum AGB values exhibit an overall increasing trend, rising from 425.750 g/m² in 2019 to 509.943 g/m² in 2024. In contrast, the minimum values display greater year-to-year variability, with markedly higher minima in 2020 and 2024-particularly in 2020, which recorded a substantial increase. The mean AGB values reflect the general trajectory of grassland biomass. From 2019 to 2020, there was a significant rise of approximately 62 g/m². After 2021, the mean value experienced a slight decline, followed by a modest rebound in 2024, suggesting relative stability in grassland biomass during this period. The standard deviation (SD) data reveal that spatial variability in AGB decreased in 2020 and remained relatively low in subsequent years. The sharp decline in SD in 2020 indicates a notable reduction in spatial heterogeneity of biomass across the region. In contrast, 2019 exhibited a relatively high SD, implying greater ecological imbalance or geographical heterogeneity, which resulted in a more uneven AGB distribution. Overall, across the four-year period, grassland AGB demonstrated moderate interannual fluctuations, characterized by a gradual increase in mean biomass and a decline in spatial variability, reflecting an overall trend of increasing biomass and improving spatial uniformity.

**Table 8:** Statistical summary of estimated grassland AGB results (g/m^2^).

Year	Maximum	Minimum	Mean	Standard Deviation
2019	425.750	23.262	133.573	72.208
2020	430.925	64.912	195.825	55.263
2021	426.817	27.488	177.679	54.627
2024	509.943	56.361	179.889	54.243

#### 3.4.2 Spatial variation

**Figure 4:**
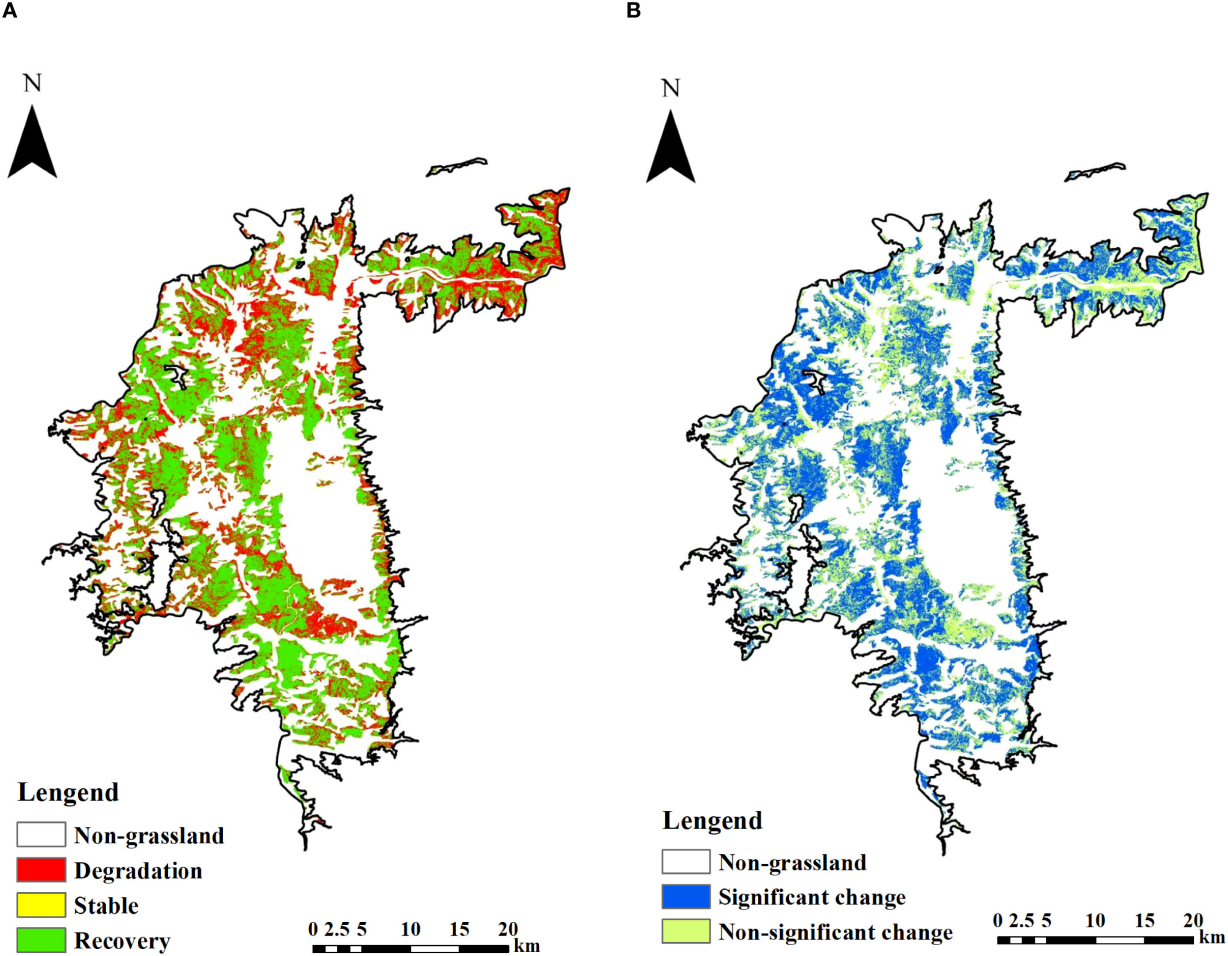
Biomass change trends and validation in the Dry-Hot Valley from 2019 to 2024. **(A)** Biomass change trend. **(B)** Significance test of biomass change trend.

Based on the biomass change trends and significance testing from 2019 to 2024 in the Yuanmou Dry-Hot Valley (**Figure 4**), the AGB of Savanna grasslands exhibits a distinct spatial gradient. As shown in **Figure 4a**, the overall trend during this period is dominated by biomass recovery. Areas of degradation are mainly located in the lower parts of the valley, concentrated at lower elevations, and are prominently distributed along river channels and valley bottoms. Stable grasslands are more uniformly distributed across the landscape, primarily situated in mid-elevation zones. In contrast, recovery zones are predominantly found at higher elevations within the valley and represent the most spatially extensive class. **Figure 4b** presents the results of the Mann-Kendall significance test for trend detection. Significant change areas (highlighted in blue) are predominantly concentrated in the northern, central, and western parts of the Dry-Hot Valley, which aligns with the spatial patterns of notable biomass increase observed in **Figure 3**. These regions correspond to areas with relatively favorable topographic and ecological conditions, as well as zones where ecological restoration measures have been intensively implemented in recent years. In contrast, non-significant change areas (shown in green) are mainly distributed in the southern and southeastern parts of the study area, where grassland productivity remained relatively stable during the observation period.

The spatial correspondence between **Figures 4a, b** is partially consistent but also reveals some mismatches that merit further interpretation. In **Figure 4a**, degraded grasslands (red) are primarily concentrated in low-altitude areas such as valley bottoms and riverbanks, indicating declining biomass trends. However, many of these areas correspond to non-significant change zones (green) in **Figure 4b**, suggesting that although a downward trend is observed, the magnitude or consistency of the change is insufficient to reach statistical significance. This mismatch may result from interannual variability in climatic conditions, such as irregular rainfall or temperature fluctuations, which can obscure clear trend signals over a relatively short time series (2019–2024). Furthermore, lowland areas are often subject to frequent land use disturbances-such as grazing, farming, or settlement expansion-leading to fluctuating biomass levels that challenge trend detection methods. In contrast, high-altitude regions showing clear recovery trends in **Figure 4a** generally align with significant change areas in **Figure 4b** (blue), reflecting effective ecological restoration or reduced anthropogenic pressure. This indicates that long-term and stable vegetation improvement, particularly in areas with favorable topography and less human disturbance, is more likely to be captured as statistically significant. These findings highlight the importance of considering both ecological processes and human activities when interpreting biomass dynamics.

## 4 Discussion

### 4.1 Estimation models for Savanna grassland aboveground biomass in the dry-hot valley

By constructing non-parametric estimation models centered on RF, GBDT, and XGBoost, and combining three feature selection methods-Spearman’s correlation, RFE, and Lasso regression-this study estimates the AGB of the Savanna grassland in the Yuanmou dry-hot valley. The results show that the RFE-RF combination outperforms all other model combinations, achieving the highest estimation accuracy and stability. The RFE algorithm recursively eliminates variables with low predictive contributions, ultimately selecting an optimal feature set with strong explanatory power. This reduces model redundancy and enhances the model’s responsiveness to key ecological factors. The key variables identified by RFE include temperature, latitude, diversity index, and certain remote sensing bands and vegetation indices-factors that are closely related to AGB in the dry-hot valley Savanna ecosystem and align well with existing research. Previous studies have highlighted that climatic factors are the primary constraints on vegetation growth in dry-hot regions. For example, Hanan et al. (Sankaran et al., 2005) pointed out that in arid savannas, temperature variations influence the effective use of water, thereby regulating the community’s net primary productivity. During the modeling process, Random Forest demonstrated its advantage in handling high-dimensional, nonlinear, and noisy remote sensing data. By randomly sampling both the data samples and variables to generate multiple diverse decision trees and using an ensemble strategy to effectively mitigate overfitting, Random Forest can stably extract the dominant factors influencing AGB variation in complex ecosystems. This method shows strong adaptability and practicality in the fragmented terrain and ecologically disturbed environment of the dry-hot valley, providing a reliable approach for high-precision remote sensing monitoring and ecological assessment of Savanna-type grasslands.

In summary, the dry-hot valley region is characterized by dramatic topographical fluctuations, diverse disturbance types, and highly heterogeneous ecosystems, with complex nonlinear interactions among variables. Traditional linear models are insufficient for accurately capturing the biomass response mechanisms in such an environment. To address this challenge, this study constructs a comprehensive variable system integrating multi-source information, including remote sensing, climate, terrain, and biodiversity. Based on the “ecological mechanism-driven” approach, this model effectively enhances its responsiveness to grassland growth conditions and improves its accuracy in representing spatial variations. On this basis, a systematic comparison of three feature selection methods and three types of ensemble models was performed. The results show that the RFE-RF model outperforms the others in estimation accuracy, robustness, and generalization ability. It effectively meets the modeling needs of the complex ecological and geomorphological landscape of the dry-hot valley, demonstrating strong regional adaptability and potential for wider application. This provides reliable technical support for the long-term monitoring and dynamic assessment of Savanna grasslands. Building upon this model, further analysis of the spatiotemporal variations of AGB will contribute to a deeper understanding of the response mechanisms of grassland ecosystems in dry-hot regions to climate and environmental changes.

### 4.2 Distribution pattern of Savanna vegetation in the dry-hot valley estimated by the model

Based on the estimation results of the optimal model, the AGB of the Savanna grassland in the Yuanmou dry-hot valley showed an overall upward trend from 2019 to 2024, reflecting the initial success of regional ecological restoration. This trend is closely linked to the ongoing implementation of grassland ecological governance policies in Yunnan Province in recent years. As a typical example of dry-hot regions, Yuanmou has long faced challenges such as land degradation and the ecological vulnerability of grasslands. To tackle these issues, the region has implemented various measures, including returning grazing lands to grassland, closed management, ecological compensation, and grassland protection subsidies and rewards, which have effectively promoted grassland resource management and ecological restoration (Yunnan Provincial Department of Agriculture and Rural Affairs, 2021). In terms of spatial distribution, AGB shows a significant gradient along elevation, with lower biomass observed in low-altitude areas, which are more heavily impacted by human disturbances such as high-intensity grazing, frequent land use, and grassland fragmentation (He et al., 2023). These elevation differences reflect spatial variations in human activity accessibility and disturbance intensity. Low-altitude areas, due to better transportation and higher land values, often become concentrated zones for agriculture and livestock, further exacerbating vegetation degradation. This, combined with the limited disturbance resistance of the Savanna ecosystem, increases the risk of degradation (Gebremedhn et al., 2023).From an ecological perspective, the Yuanmou dry-hot valley is located in a typical arid climate zone, characterized by low annual precipitation and uneven seasonal distribution, which exacerbates water stress on grassland vegetation. Moreover, the Savanna ecosystem, being a transitional type between herbaceous and shrubland vegetation, is highly sensitive to water-heat conditions, terrain factors, and human disturbances. While initial management efforts have yielded some positive results, a lack of sustained investment and monitoring mechanisms may undermine the stability of ecological restoration. Therefore, it is recommended to establish a multi-scale monitoring system, strengthen the integration of remote sensing and ground surveys, and promote a comprehensive grassland management model that combines policies, technology, and management efforts.

In summary, the spatiotemporal dynamics of biomass in the Savanna grassland of the Yuanmou dry-hot valley not only reflect the positive impacts of ecological policies but also highlight the conflicts between grassland utilization and conservation in low-altitude areas. Moving forward, it is crucial to enhance grassland classification and regulation, implement zoned management, and establish long-term monitoring systems. This will support the transition from extensive utilization to more refined management, ultimately improving the sustainability of ecosystem services in dry-hot regions.

### 4.3 Limitations and future directions

Developing high-precision models for grassland biomass estimation is essential for the effective monitoring and sustainable management of grassland ecosystems. However, the accuracy of biomass estimates is strongly influenced by both the quality of multi-source data and the strategies used for their integration. For instance, temporal discontinuities caused by missing annual data may limit a model’s ability to capture interannual variability in grassland productivity. In addition, the uneven temporal distribution of field samples can lead to overfitting or underfitting during model calibration. A key challenge, therefore, is to establish a methodological framework that not only identifies and retains the most informative variables but also effectively conveys their ecological relevance to appropriate machine learning algorithms. To address this, the present study introduces an integrated modeling framework that combines multiple feature selection methods with a suite of machine learning models for biomass estimation in ecologically heterogeneous regions. To mitigate the impact of sample imbalance, we applied techniques such as data standardization and proportional partitioning of field samples into randomized training and testing sets. These approaches substantially improved the transferability and generalizability of the optimized models across regions with diverse ecological conditions. Looking ahead, future research should focus on further integrating multi-source remote sensing datasets—including hyperspectral, microwave, and LiDAR—and enhancing their compatibility with process-based ecological models. Such efforts are expected to strengthen the robustness and expand the applicability of high-precision biomass estimation frameworks across complex grassland landscapes.

## 5 Conclusion

This study developed a remote sensing-based modeling framework specifically tailored to the unique ecological characteristics of the savanna grassland ecosystem in the Yuanmou dry-hot valley. Unlike conventional approaches that often rely on a single vegetation index or a single modeling technique, our framework addresses the complexity and heterogeneity inherent in dry-hot valley ecosystems. A comprehensive, multidimensional feature set was constructed, incorporating five categories of key ecological drivers: remote sensing spectral bands, vegetation indices, diversity indices, topographic factors, and climatic variables. This integrative feature set captures the diverse environmental conditions and ecological gradients influencing AGB dynamics in the region. To enhance model performance and reduce redundancy, three feature selection techniques-Spearman’s rank correlation, RFE, and Lasso regression-were combined with three ensemble learning models: RF, GBDT and XGBoost. This combination yielded nine modeling pathways, which were systematically organized into a reproducible workflow: Feature Selection → Model Construction → Accuracy Evaluation. Model assessment results indicated that the RFE-RF model performed best under the highly heterogeneous, topographically complex, and disturbance-prone conditions of the dry-hot valley. It achieved an R² of 0.6975, an RMSE of 89.3436 g/m², and an MRE of 0.7282, suggesting strong robustness and generalization capability. These results underscore its suitability for biomass estimation in arid to semi-arid environments and ecologically fragile transition zones characterized by spatial complexity and nonlinear ecological responses. Based on model outputs, AGB in the savanna grasslands showed a consistent upward trend between 2019 and 2024, with annual mean values of 133.573 g/m² (2019), 195.825 g/m² (2020), 177.679 g/m² (2021), and 179.889 g/m² (2024). Although a slight decline was observed in 2021 compared to 2020, the overall biomass level remained relatively high, indicating that grassland productivity has improved under the continued implementation of ecological management strategies across the region. In terms of spatial patterns, grassland dynamics in the study area were predominantly characterized by recovery trends, particularly in the high-altitude zones of the western and northern regions. These areas exhibited both notable increases in aboveground biomass and statistically significant change, likely driven by favorable topographic conditions and the implementation of ecological restoration measures. In contrast, degraded grasslands were mainly distributed in low-lying river valleys and valley bottoms in the southern and eastern parts of the study area. However, many of these degradation zones did not exhibit statistically significant trends, suggesting high interannual variability and frequent anthropogenic disturbances, such as grazing or land conversion, that mask long-term patterns. Stable grasslands were distributed relatively evenly across mid-altitude regions, reflecting localized ecological resilience under moderate environmental stress. These findings emphasize the potential for natural ecological recovery in environmentally fragile regions-particularly when supported by targeted management interventions, long-term monitoring, and integrated modeling strategies.

## Data Availability

The original contributions presented in the study are included in the article/supplementary material. Further inquiries can be directed to the corresponding author/s.
